# High-order brain interactions in ketamine during rest and task: A double-blinded cross-over design using portable EEG

**DOI:** 10.21203/rs.3.rs-3954073/v1

**Published:** 2024-03-21

**Authors:** Agustin Ibanez, Ruben Herzog, Florentine Barbey, Md Nurul Islam, Laura Rueda-Delgado, Hugh Nolan, Pavel Prado, Marina Krylova, Nooshin Javaheripour, Lena Danyeli, Zümrüt Sen, Martin Walter, Patricio Odonnell, Derek Buhl, Brian Murphy, Igor Izyurov

**Affiliations:** Trinity College Dublin; University Hospital Jena; Jena University Hospital; Jena University Hospital; Sage Therapeutics

## Abstract

In a double-blinded cross-over design, 30 adults (mean age = 25.57, SD = 3.74; all male) were administered racemic ketamine and compared against saline infusion as a control. Both task-driven (auditory oddball paradigm) and resting-state EEG were recorded. HOI were computed using advanced multivariate information theory tools, allowing us to quantify nonlinear statistical dependencies between all possible electrode combinations. Results: Ketamine increased redundancy in brain dynamics, most significantly in the alpha frequency band. Redundancy was more evident during the resting state, associated with a shift in conscious states towards more dissociative tendencies. Furthermore, in the task-driven context (auditory oddball), the impact of ketamine on redundancy was more significant for predictable (standard stimuli) compared to deviant ones. Finally, associations were observed between ketamine’s HOI and experiences of derealization. Conclusions: Ketamine appears to increase redundancy and genuine HOI across metrics, suggesting these effects correlate with consciousness alterations towards dissociation. HOI represents an innovative method to combine all signal spatial interactions obtained from low-density dry EEG in drug interventions, as it is the only approach that exploits all possible combinations from different electrodes. This research emphasizes the potential of complexity measures coupled with portable EEG devices in monitoring shifts in consciousness, especially when paired with low-density configurations, paving the way for better understanding and monitoring of pharmacological-induced changes.

## 1 Introduction

Ketamine, a non-competitive N-Methyl-d-aspartate (NMDA) receptor antagonist considered a non-serotonergic psychedelic compound, has garnered attention for its capacity to induce alterations in the global dynamics of conscious states^1,2^. It holds promising implications for pharmacological interventions, especially in the treatment of depression and other mood-related disorders^3,4^. Specifically, it has been linked to experiences of derealization (i.e., feeling detached from surroundings), depersonalization (i.e., feeling detached from self), and altered perception of the body, environment, and time^1,2^. Various studies employing behavioral analyses and neuroimaging have illustrated shifts in neural patterns during ketamine administration (see reviews:^5,6^). Although ketamine does not directly target serotonergic receptors like classical psychedelics, evidence from neuroimaging and electrophysiological studies suggest common signatures and potential mechanisms of altered states of consciousness^7–9^. Both ketamine and serotonergic psychedelics have been linked to modulations of the Default Mode Network (DMN)^10^ as well as increases of brain entropy^7,11^. Many of the brain pattern shifts observed under ketamine and psychedelics entail a decrease in top-down brain organization paired with an enhanced emphasis on the lower hierarchies of sensory information^12–15^. These observations are consistent with the Relaxed Beliefs Under Psychedelics (REBUS) model proposed by Carhart-Harris and Friston, which suggests that psychedelics alter consciousness by reducing the weight of prior beliefs when processing bottom-up sensory information emerging from the periphery^15^. The reduced top-down control from higher brain hierarchies results in a richer conscious experience and sometimes prediction errors manifesting as perceptual illusions or experiences of dissociation^12,15^. During the ketamine experience, this dissociation may be further evidenced through complexity measures of brain activity, as reflected in increased entropy^15–18^.

Emergent innovations in clinical trials emphasize the adoption of portable dry electrodes, addressing a significant gap in the demand for accessible, reliable, and economical biomarkers to monitor drug effects^19,20^. However, studies exploring the effects of ketamine with low-density electrodes are scarce. An innovative and robust approach to probe into these effects in low-density setups encompasses brain high-order interactions (HOI)^21^. Three salient features made HOI critically relevant^22–26^. First, as opposed to standard event-related potentials (ERP), oscillations, and connectivity metrics, HOI can compute all possible interactions between signals (here, electrodes). Despite the advantages of having high-density arrays (as source localization, increased spatial resolution, and interpolation of bad electrodes), the HOI approach becomes computationally challenging in these arrays, inducing a selection bias imposed by dimensionality reduction techniques. In contrast, despite their reduced spatial resolution, low-density arrays allow us to feasibly compute all potential interactions, thereby maximizing information conveyance—a feature unmatched by any other technique. Thus, leveraging HOI provides a unique and novel improvement to understanding brain dynamics using low-density arrays, which usually reduce data’s granularity. Second, HOI effectively captures the global dynamics of brain organization^22–26^, a critical component of different consciousness states (disorder of consciousness, anesthesia, transitions, and conscious access^27–31^), making them ideal for elucidating the effects of pharmacology. Furthermore, the evidence underscores HOI’s superior robustness when compared to traditional connectivity metrics across various modalities, including fMRI and EEG, and across different brain conditions^23–26^. In brief, when combined with entropy measures and low-density EEG, HOI may constitute a crucial approach for assessing global dynamic changes induced by ketamine and other drugs used in clinical trials settings.

**Figure 1:**
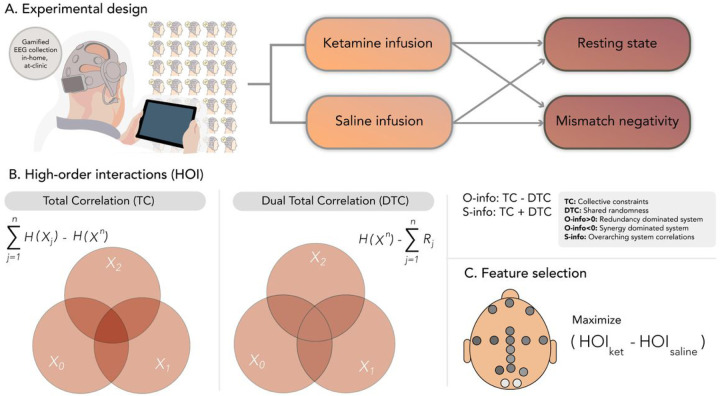
Overview of experimental design and data analysis. **A).** Subjects participated in a double-blind crossover design using portable EEG, capturing both resting states and task-based recordings (namely, a gamified oddball paradigm inducing a typical mismatch negativity). In randomized sessions, participants received both ketamine and saline infusions. **B).** Analysis of high-order interactions (HOI) entailed measurements of total correlation (TC) and dual total correlation (DTC), O-information and S-information (see Methods). **C).** Feature selection across the two designs (rest and task) was employed to pinpoint the primary differences between the ketamine and saline conditions.

The current study (Fig. 1) investigated changes in HOI during a drug-induced altered state of consciousness, adopting a double-blinded cross-over design, enrolling 30 adults (but only 29 were used in this analysis), and administering racemic ketamine via a continuous infusion protocol (Fig. 1A). We juxtapose the effects of ketamine against a saline infusion as a control, leveraging both task-driven (gamified auditory oddball paradigm) and resting-state EEG sampling methodologies^32,33^. Using a low-density, wireless, dry electrode EEG system^34^, participants’ responses to ketamine were captured. Alongside EEG data, the subjective effects of ketamine administration were measured through self-reported and clinician-administered questionnaires. Our analyses focused on HOI assessed with entropy-based measures^35^. We predicted that HOI would yield consistent results across designs (resting state, task) and conditions (ketamine vs saline and deviant vs. standard stimuli in the task). We expected that effects would mainly be observed in an alpha band which is systematically reported to be altered in ketamine studies^32,33^. Such effects are anticipated to be more pronounced during resting state than in task^36^, given ketamine’s hypothesized role in diminishing top-down control, manifesting as redundancy of bottom-up signals in brain dynamics^15^. To assess this specific hypothesis of increased redundancy associated with the reduction of top-down hierarchical control, we include other measures of global dynamics (see below). Furthermore, during task-driven conditions, particularly a passive and gamified auditory oddball paradigm, the more predictable stimuli (i.e., standard) are expected to yield similar increased effects in contrast to the deviant stimuli, as prediction may be more impacted by the decreased top-down influences. Finally, we assessed whether HOI-associated changes during ketamine administration correlate with subjective consciousness-altered states in terms of dissociative states (i.e., derealization scores)^37–39^. In brief, this study aims to establish the ability of a portable, low-density dry EEG device to capture the overarching global brain dynamics of ketamine-induced shifts, both in resting and task-engaged states, serving as potential brain signatures of altered states of consciousness.

## 2 Methods

EEG data (16 electrodes, Cumulus Neuroscience dry-sensor 16 electrode headset) were recorded during a resting state and a mismatch negativity task under saline or ketamine injection (Fig. 1A). A pipeline based on multivariate information theory was applied to investigate whether saline could be discriminated against ketamine under both rest and task. The complete set of possible combinations between electrodes at all orders of interactions (from 2 to 16) was assessed (Fig. 1B–C). The EEG used here has been presented previously on reference^40^, where a detailed description can be found.

### 2.1 Participants

Study participants (N = 30 males, 25.57 +/− 3.74y) were carefully selected to ensure a consistent and controlled environment for the research. To qualify, individuals had to be males aged between 18 and 55. Women were excluded to avoid undetected pregnancy risk and to reduce sample variability. Participants’ health was critically assessed through a physical examination, medical history, vital signs, a 12-lead Electrocardiogram (ECG), and clinical laboratory tests. Participants were excluded if they had a current or past history of psychiatric disorders as per the ICD-10, especially those with a history of drug or alcohol dependence/abuse in the last 6 months. Furthermore, any serious unstable illnesses, including but not limited to hepatic, renal, respiratory, cardiovascular, endocrine, and neurological disorders, led to exclusion. This also encompassed subjects with unresolved seizure causes, conditions that likely alter brain morphology or physiology such as uncontrolled hypertension or diabetes, significant acute illnesses a week before the drug administration, notable history of drug or food allergies, and the use of specific medications including antidepressants, anti-psychotics, anxiolytics, and others. Additionally, color-blind individuals were also not considered for this study. Each participant’s commitment to the study’s guidelines and restrictions was crucial, and they had to demonstrate their understanding and willingness to participate. This study was approved by the Institutional Review Board of the Otto-von-Guericke University Magdeburg, Germany, and informed consent was obtained from all participants (Approbation code number: 123/18).

### 2.2 Study Design

The study was a saline-controlled, double-blind, randomized, crossover study with healthy participants in their homes, designed to investigate the acute and delayed effects of ketamine on EEG and behavioral measures. Participants were invited to the laboratory to complete repeated measurements: at enrollment, on the days of infusion of ketamine or saline administration (randomized across two visits), and on the days after infusion (Fig. 1). The two infusion days occurred four weeks apart following the same study protocol. All EEG recordings were performed with the portable dry EEG system developed by Cumulus Neuroscience (www.cumulusneuro.com).

### 2.3 Ketamine infusion

During the infusion, participants were seated in a comfortable chair, and their legs were elevated on a footrest. Overall mobility was restricted as a cannula was placed in each arm; one for the delivery of ketamine or saline and the other to draw blood samples. Participants were administered a single IV infusion (of a total volume of 50 ml) of 0.5 mg/kg of racemic ketamine hydrochloride over 40 mins or IV saline solution (0.9%) over 40 mins. During the infusions, the tablet was mounted on a tablet holder and operated by the researchers as participants could not bend their arms at that time.

## 2.4 EEG recordings

EEG data was collected using the wireless 16-channel dry sensor EEG system developed by Cumulus Neuroscience (Cumulus, www.cumulusneuro.com), suitable for use in various supervised and unsupervised settings^34^. The analog front-end is based on the ADS1299 chip-set from Texas Instruments, incorporating a high input impedance of 1GΩ, a configurable driven bias function for common-mode rejection, built-in impedance checking, and configurable gain and sampling rates. The left mastoid is used for reference and the right mastoid for driven-bias, with single-use, snap-on electrodes attached to wires extending from the headset. An onboard processor and Bluetooth module transmit 250Hz EEG data to another device (an Android tablet in this case), transferring it to a cloud server for storage and processing. Flexible Ag/AgCl coated polymer sensors of a comb-design (ANT-Neuro/eemagine GmbH) are used to achieve a stable and dermatologically safe contact to the scalp through the hair. The electronics and sensors are mounted on a flexible neoprene net for comfort and ease of montage. Incorporating natural landmarks in the headset form factor and the stretchable structure enable consistent placement by non-experts in line with the 10–20 sensor system.

### 2.5 Resting-state session

Eyes-closed resting state EEG recordings were collected during the first 20 mins after either saline or ketamine infusion. Participants were instructed to rest with closed eyes and remain still during the recording. For the resting state, the first 10 minutes post-injection were not considered in the analysis to discard transients associated with injection, yielding 10 minutes of stable post-injection resting state performed at home.

### 2.6 Passive-listening auditory oddball paradigm

During the last 15 minutes of each infusion, we used an app-based version of the passive auditory oddball task developed by Cumulus – Sonic Scenes – eliciting the Mismatch Negativity (MMN) of infrequent ‘deviant’ stimuli in a train of ‘standard’ stimuli. The task was performed passively – the subject merely needed to remain still while listening to repetitive auditory stimuli. The participant was prompted to put on headphones and adjust the volume until he could clearly hear some sample tones. When ready, the participant asked to press play on a silent movie, which lasted 15 mins. Tones played throughout while the participant watched the film. Eight short films were used in an arbitrary cycled order, each consisting of silent clip montages compiled from stock footage. There were no narratives or subtitles. Each movie session incorporated 1000 ‘standard’ tones (100ms; 1000Hz) and 200 pitch deviants (100ms; 1200Hz).

### 2.7 Subjective scores of dissociative states

We assessed the Clinician-Administered Dissociative States Scale (CADSS), which measures dissociative states^41^, and the 5D-ABZ, which assesses self-reported altered states of consciousness^42^. CADSS has 3 subscales (“Amnesia”, “Derealization”, “Depersonalization”), while 5D-ASC entails five dimensions (“Oceanic Boundlessness”, “Dread of Ego Dissolution (DED)”, “Visionary Restructuralization”, “Auditory Alterations”, “Vigilance Reduction“). Both questionnaires were administered in the laboratory 1h before and 1.5hr after the infusion. The scores’ difference between before and after infusion were used to rate the subjective changes for both saline and ketamine infusion. All questionnaires used validated German-language versions, except for the CADSS questionnaire translated by the research team as no validated translation was available when the study was conducted.

### 2.8 EEG data preprocessing

Using Cumulus’ proprietary algorithms, EEG data were pre-processed to correct the integrity of timing information and eye-movement artifacts. For the oddball paradigm, baseline-adjusted EEG data were band-filtered between 0.25–40 Hz and analyzed in 30-s epochs. Corrective procedures are applied for epochs with missing and anomalous data, including eye and movement artifacts. To preserve the same number of channels (16) during the whole analysis, time points from all channels were removed if at least one channel had a flat signal in those points, which was required to explore all the possible high-order interactions. One subject was removed from further resting state analysis because there was < 1s of valid data in the ketamine condition. No differences in the number of selected points were found after the artifact removal (Supplementary Tables 1 and 2) across conditions (ketamine/saline) and recordings (rest/task). Resting-state data were bandpass filtered in the canonical EEG frequency bands: δ: 0.5–4 Hz, θ: 4–8 Hz, α: 8–12 Hz, β: 12–30 Hz, γ: 30–40 Hz). Given that our goal was not to compute ERPs, we pooled all the valid trials (with at least 125ms valid signal) corresponding to the deviant and standard tones separately to perform the analysis. In the following analysis, the 5 bands plus broadband (0.5–40 Hz) data was used for the resting state, while for the tasks only broadband data were used to focus only on the evoked rather than the induced response. EEG data obtained appeared reliable as the standard effects on ketamine vs. saline solution were noticeable in the EEG spectrum (across the alpha peak and typical frequency/power decay, see Figure S1).

### 2.9 Pairwise and high-order interactions

To assess the hypothesis of ketamine-induced specific effects of redundancy in brain dynamics, different measures of entropy characterizing different properties of brain dynamics were computed. We used tools from multivariate information theory i) to quantify the nonlinear statistical dependencies between all the possible combinations between electrodes and ii) to distinguish the nature of these dependencies in terms of collective constraints (total correlation, TC), shared randomness (dual total correlation, DTC), synergy (O-info < 0), redundancy (O-info > 0), and overarching correlations (S-info) (see reference^35^ for a detailed explanation of these measures).

Consider a system of *n* random variables denoted by X^n^ = (X_1_, … ,X_n_). The TC, DTC, O-information (O), and S-information (S) are generalizations of the mutual information (MI)^35^, which can be respectively expressed as:

1 $$TC\left(X^{n}\right)=\sum_{i=1}^{n}H\left(X_{i}\right)-H\left(X_{1},\ldots ,X_{n}\right)$$

2 $$DTC\left(X^{n}\right)=H\left(X_{1},\ldots ,X_{n}\right)-\sum_{i=1}^{n}H\left(X_{i}|X_{-i}^{n}\right)$$

3 $$O\left(X^{n}\right)=TC\left(X^{n}\right)-DTC\left(X^{n}\right)$$

4 $$S\left(X^{n}\right)=TC\left(X^{n}+DTC\left(X^{n}\right)\right)$$

where *H(X*_1_*, …, X*_*n*_) is the joint Shannon’s entropy of the n variables, *H*(*X*_*i*_) the entropy of the i-th region H *(X*_*i*_*|X*^*n*^_*−i*_) is the entropy of the i-th region conditioned by the activity of the whole system without it – which is known as “residual entropy,” and is denoted as *R*_*i*_. Above, X_−_i^n^ represents the vector of all variables except X_i_, i.e., (X_1_, …, X_i−1_, X_i+1_, …, X_n_). Estimations were performed using the Gaussian copula approximation^25,43^. As for n = 2 TC = DTC = mutual information, only the TC was computed for each possible pair of electoreds. For the high-order interactions (from 3 to 16) all metrics were computed for each possible combination of electrodes at each order of interaction (Fig. 1B and C).

Broadband (0.5–40 Hz) and filtered EEG signals (δ: 0.5–4 Hz, θ: 4–8 Hz, α: 8–12 Hz, β: 12–30 Hz, γ: 30– 40 Hz) were analyzed considering all the possible combinations of electrodes at each order of interaction, here denoted by **k** (120 interactions for **k** = 2, 560 for **k** = 3, 1.820 for **k** = 4, 4.368 for **k** = 5, 8.008 for **k** = 6, 11.440 for **k** = 7, 12.870 for **k** = 8, 11.440 for **k** = 9, 8.008 for **k** = 10, 4.368 for **k** = 11, 1.820 for **k** = 12, 560 for **k** = 13, 120 for **k** = 14, 16 for **k** = 15 and 1 for **k** = 16). An *n-plet* represents a particular combination of n electrodes, and the effect of ketamine on it was assessed via the effect sizes.

### 2.10 Effect sizes

To characterize the size of the effect associated with each measurement, we used Cohen’s *d* effect size for paired samples^44^:

(5), $$d=\frac{\mu_{ket}-\mu_{sal}}{s}$$

where *μ*_*ket*_ and *μ*_*sal*_ are the average measure of the ketamine and saline condition, respectively, and *s* is the standard deviation of the difference of means (i.e *μ*_*ket*_ *– μ*_*sal*_). This metric measures a standardized mean difference between paired samples, and its sign indicates the direction of the effect, i.e. if d > 0 means that ketamine increases the measure.

### 2.11 Statistical analyses

For the selected features, a non-parametric Wilcoxon sign rank test for paired samples with the False Discovery Rate (FDR) correction for multiple comparisons was performed. As in previous work on HIOs^22–26^, the statistical correction was not directly assessed for each HOI given the non-selective data approach, including all interactions^45^. Conversely, we used Cohen’s D to report the effect size of HOI^22,24,25^ as *p*-values can be artificially inflated. To compute the association between HOI and subjective scores, the change (ketamine - saline) in HOI was (Pearson) correlated with the change in subjective scores, yielding one R2 per n-plet and subjective score. We evaluated the significance of associations by a permutation test followed by post-hoc FDR correction for multiple comparisons. First, we generated a null distribution of R2 values by randomly permuting the correspondence between HOI and subjective scores 1000 times. Only correlations values above the 99.9-th percentile of their respective null distribution were submitted to FDR correction (p < 0.005).

## 3 Results

### 3.1 Ketamine increases redundancy in the alpha band during resting state

**Figure 2:**
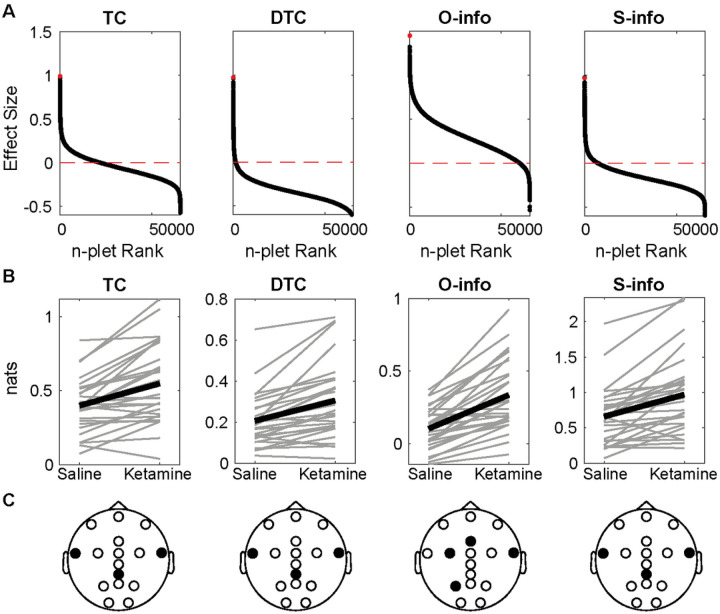
Ketamine increases redundancy in the resting state alpha band. **A)**Each panel shows the effect sizes of each n-plet (i.e. each possible combination of electrodes from 2 to 16) sorted in decreasing order, with a red horizontal line showing the 0. The red dot denotes the maximum effect size. **B)** The n-plet with the largest effect under saline and ketamine for each subject (gray) and for the average (black). Only O and S-info yielded FDR-corrected Wilcoxon sign rank p-values<0.001. **C)** EEG layout with the electrodes composing the n-plet in black.

Results evidenced positive and negative effect sizes for all the combinations between filtering bands and metrics. The alpha band showed the largest (metric increase with ketamine) and smallest effect sizes (metric decrease with ketamine) for all the metrics (Fig. 2A and S1). Indeed, only O-info and S-info significantly increased in the alpha band (Fig. 1B, Wilcoxon sign rank test, p < 0.001 after FDR correction). Among the 4 metrics in the alpha band, the largest increase was found for the O-info, which included the P3, FCC3, Fz, and FT8 electrodes (Fig. 2C). The S-info was related to the CPz, FT7, and FT8 electrodes. Despite the other filtering bands also exhibiting a marked tendency both for increases and decreases, no significant effect was found (Figure S2). These results indicate that the effect of ketamine can be better explained as an increase in the overarching correlations –Specifically redundancy– between temporal and parietal recordings in the alpha band during the resting state.

### 3.2 Ketamine increases redundancy for predictable stimuli

**Figure 3:**
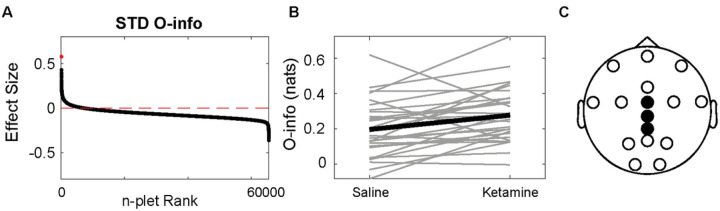
Redundancy increases in the standard tone for mismatch negativity task. **A)** Effect sizes of each n-plet for the oddball paradigm, sorted in decreasing order, with a red horizontal line showing the 0. The red dot denotes the maximum effect size **B)** The n-plet with the largest effect under saline and ketamine for each subject (gray) and for the average (black). **C)** The electrodes involved in the best feature. Wilcoxon sign rank FDR-corrected p-value<0.005.

We found both positive and negative effect sizes for all the combinations between metrics and stimuli type (STD, standard; DEV, deviant) for the auditory oddball task (Fig. 3A, S4). We compared the STD and DEV response under saline and under ketamine, without comparing the STD to the DEV tone. The deviant tone showed slightly larger absolute effect sizes. Significant differences were found only for the O-info in the standard tone (Fig. 3B, FDR-corrected Wilcoxon sign rank test, p < 0.005), which included CPz, Cz and FCz electrodes. The results for the rest of the metrics and the deviant tones are shown in Figure S4. The same set of electrodes was found for both conditions in the tasks. These results indicate that ketamine significantly increases the evoked responses for predictable but not deviant stimuli.

### 3.3 Derealization correlates with changes in resting state theta-band high-order interactions

**Figure 4:**
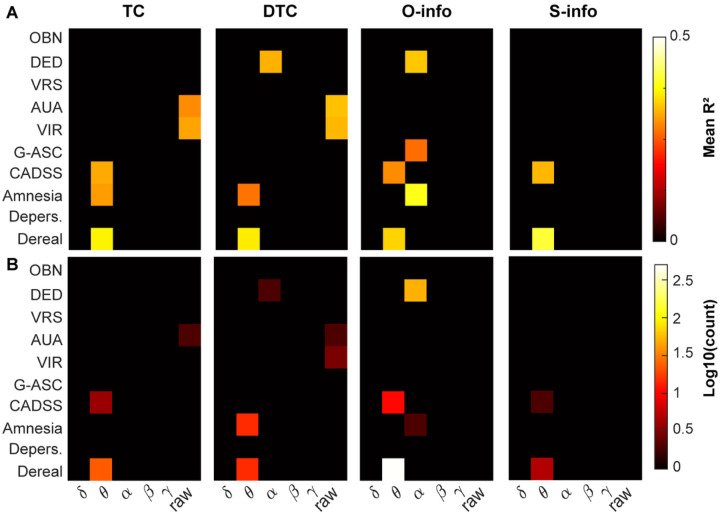
Association between subjective scores and HOI. A) Average R2 only for the significant (FDR, p<0.005) associations between the change in each dimension of subjective score (see Methods) and change in HOI. B) Same as A, but showing the number of significant HOI in log10 scale to improve visualization.

Finally, we investigated the association between the change in resting state HOI and the change in subjective scores, as measured via the CADSS and the 5D-ASC (see Methods). We found the largest number of significant correlations for the O-info in the theta band and CADSS Derealization score, followed by the alpha band in the ‘Dread of ego dissolution’ item (Fig. 4B). Indeed, the theta band showed the largest association values for all the subjective scores, followed by the alpha band (Figure S5). The presence of the alpha band is consistent with results of the previous section, where the largest effect sizes were found in the alpha-band.

This finding suggests that changes in the alpha band are not only indicative of the change in the global state of consciousness, but also of more subtler aspects of the experience. In turn, changes in the theta band HOI may be indicative of the level of derealization experienced by the subjects following ketamine infusion, as compared to saline.

## 4 Discussion

This study aimed to investigate the neuropharmacological effects of ketamine on consciousness, employing a novel and robust approach using HOI, with portable EEG in a double-blinded cross-over design. This exploration centered around low-density arrays, addressing a current gap in research methodology. Key findings indicate that ketamine administration induced a significant increase in the correlations and redundancy of brain dynamics, particularly evident in the alpha frequency band, consistent with observations across classical EEG studies of ketamine. Results open a new avenue for future studies using portable, low-density recordings captured during pharmacological interventions that maximize the combined information across electrodes.

Our findings bolster the application of HOI for low-density arrays. The shifts in brain dynamics, Specifically an increase in redundancy, are most pronounced in alpha (echoing previous reports focused on this band, tasks and rest^46–48^). Such alterations, particularly in the resting state, support the potential of ketamine to decrease top-down control and increase the sensitization to bottom-up signals^1,2,12–15^. This phenomenon is manifested by an upswing in redundancy within brain dynamics, possibly reflecting a reduced top-down inhibition and an amplification of lower hierarchies of sensory information^12–15^. The increase in redundancy following ketamine administration was larger during resting states than tasks, suggesting the spontaneous shift of the conscious state towards less controlled states during resting state, as dissociation. Also, the app-based auditory oddball paradigm revealed that the effects on redundancy are more conspicuous for predictable (standard) stimuli than for deviant stimuli, accentuating ketamine’s influence on spontaneous, less controlled cognitive processes^12,15^, as low-level stimuli prediction. Furthermore, a significant correlation emerged between the effects of ketamine on resting-state HOI in the alpha and theta band and the subjective experiences of DED and derealization, respectively, both core facets of ketamine-induced dissociative states. DED refers to a change in the perception of selfhood and subject-object boundaries, while derealization is the feeling of disconnection from the surroundings^12,15,16^. These results confirm reported associations between the alpha-band and ´*ego-integrity’* 49and derealization measures and theta band^50,51^, particularly observed with ketamine^12^. Our findings support the idea that subjective alterations in consciousness are anchored in changes in brain dynamics and functional organization and thus can be properly tracked with measures of neural complexity^52^.

This study has limitations that open different avenues for further research. Compared to high-density electrodes, low-density dry electrodes offer limited information and have a lower signal-to-noise ratio. However, we attempted to mitigate these shortcomings with several internal controls in our design, including (a) no significant difference in the number of artifacts across conditions; (b) using a robust double-blinded cross-over design, which minimized individual heterogeneity, variance, and the distribution of random effects from any confounding variables across conditions; (c) verification of the expected effects of ketamine on the power spectrum (supplementary data). As a result, we found a systematic directional consistency of the effects of HOI across various measures. Moreover, since the primary HOI differentiating the ketamine vs. saline conditions did not involve frontal areas, the results reduce the possibility that differences in eye movement across conditions could account for these effects. Despite these measures, our findings require further validation using high-density electrode arrays, which might entail reducing the number of HOI. Exploring other brain measures like fMRI, where HOI can be assessed, would also be beneficial. While our sample size was modest, it remains comparable to, or even larger than, similar studies in the field (see reviews:^5,6,12,13^). In any case, future studies must replicate the findings in larger samples. This study only included male participants, so the effects of gender and the potential particular changes observed in women require further investigation. Lastly, our study does not explore brain dynamics in correlation with plasma measurements of ketamine and potential whole-body effects, such as cardio-dynamics and sensorimotor activity. These aspects offer promising avenues for future research.

## 5 Conclusions

Our results suggest that HOI provides a novel approach to maximizing the information obtained from low-density EEG in pharmacological interventions. More Specifically, ketamine fosters an increase in redundancy and HOI across measures suggesting changes in how the brain processes information, leading towards dissociation. Further research is needed to evaluate the potential of HOI to track the potential therapeutic effect of ketamine for psychiatric diseases as depression. These effects offer a deeper understanding of the neuropharmacological actions of ketamine and underscore the potential of portable EEG devices in charting alterations in consciousness, especially when combined with the HOI in low-density setups. This could lay the foundation for future endeavors aimed at better capturing the subject’s preparedness and the subsequent pharmacological-induced changes in therapeutic settings across neuropsychiatric conditions.
